# Plant polyamines in stress and development: an emerging area of research in plant sciences

**DOI:** 10.3389/fpls.2014.00319

**Published:** 2014-07-03

**Authors:** Rubén Alcázar, Antonio F. Tiburcio

**Affiliations:** Unitat de Fisiologia Vegetal, Departament de Productes Naturals, Biologia Vegetal i Edafologia, Facultat de Farmàcia, Universitat de BarcelonaBarcelona, Spain

**Keywords:** polyamines, putrescine, spermidine, spermine, thermospermine, transglutaminase, stress, ROS

Compelling evidence indicates the participation of polyamines in abiotic and biotic stress responses in plants. Indeed, genetic engineering of polyamine levels in plants has successfully improved biotic and abiotic stress resistance in model plants and crops. We anticipate that many of the current challenges in agriculture to cope with climate change and maintain nutritional quality of fruits and vegetables can be approached by considering the polyamine pathway.

The polyamine field is very dynamic as demonstrated in the large number of monthly publications in all disciplines studying polyamines (including plant sciences, human health, and microbiology). It is composed by a broad spectrum of research laboratories spread around the world, which have provided important contributions into mechanistic processes, present and future practical applications. Still, some areas remain to be explored which makes this a fascinating topic in plant sciences. In this topic, the Editors aimed at establishing a broad perspective of polyamine action in plant stress and development by inviting key researchers in the field. We would like to thank all contributors for joining us in this special topic in Frontiers in Plant Science and we hope that authors have enjoyed the interactions and discussions with editors and reviewers around their excellent works.

This topic contains five reviews, five original research studies and one hypothesis and theory article. Minocha et al. (2014) provides a review update about the complex relationship between polyamines and abiotic stress tolerance with selected examples of polyamine genetic engineering that improve tolerance traits, the concept of stress priming and interactions of polyamines with ROS and other signaling pathways. Do et al. (2014) analyze the polyamine transcriptome and metabolome in rice cultivars differing in salt tolerance, which provides an interesting comparison with potential applications in plant breeding. The interactions between biotic stress and polyamines are reviewed by Jiménez-Bremont et al. (2014) who synthesizes the current knowledge of polyamine metabolism in compatible and incompatible interactions, discusses about the capacity of phytopathogenic microbes of modulating polyamine metabolism for their own benefit, interactions with beneficial microorganisms and practical applications to induce biotic stress tolerance. Marco et al. (2014) reports that overexpression of *SAMDC1* enhances the expression of defense-related genes in *Arabidopsis* and promotes disease resistance against bacterial and oomycete pathogens. Another complementary perspective, Valdés-Santiago and Ruiz-Herrera (2014) provide an original and illustrative view on recent advances about polyamine metabolism in fungi, ranging from mutant characterization to potential mechanisms of action in response to various stresses in selected fungal models. Although free polyamines often capture most of our attention, polyamines are present in free and bound forms resulting from interactions with cellular macromolecules. Some of these interactions occur by covalent linkages with specific proteins in reactions catalyzed by transglutaminases (TGase). Del Duca et al. (2014) provide an original review about the role of TGase on senescence and cell death in various plant models. Interestingly, the role of plant TGase is mediated by a similar molecular mechanism described for apoptosis in animal cells, which opens an interesting field for further exploration in the future. In the context of mechanistic processes, accumulating evidence suggests that polyamines play essential roles in the regulation of plant membrane transport. The review by Pottosin and Shabala (2014) summarizes the effects of polyamines and their catabolites (i.e., ROS) on cation transport across plant membranes, and discuss the implications of these effects for ion homeostasis, signal-transduction, and adaptive responses of plants to environmental stimuli. The regulation of ROS homeostasis by the polyamine back-conversion pathway catalyzed by polyamine oxidase 3 (PAO3) has been investigated by Andronis et al. (2014) in an original article. From a developmental perspective, Tong et al. (2014) provide evidence for the modulation of auxin signaling by thermospermine, which sheds light into polyamine mechanisms of action on plant development. In ripening apple fruit, Deyman et al. (2014) report the interaction of polyamines with products of polyamine catabolism (i.e., GABA). Traditionally, polyamines are described as organic polycations, when in fact they are bases that can be found in a charged or uncharged form. Although uncharged forms represent less than 0.1% of the total polyamine pool, Ioannidis and Kotzabasis (2014) propose that the physiological role of uncharged polyamines could be crucial in chemiosmosis. The authors explain the theory behind polyamine pumping and ion trapping in acidic compartments (i.e., the lumen of chloroplast) and how this regulatory process could improve either photochemical efficiency and the synthesis of ATP or fine tune antenna regulation and make plants more tolerant to stress.

## Conflict of interest statement

The authors declare that the research was conducted in the absence of any commercial or financial relationships that could be construed as a potential conflict of interest.
